# Spontaneous Rectus Sheath Abscess in an Intravenous Drug User

**DOI:** 10.7759/cureus.9009

**Published:** 2020-07-05

**Authors:** Aveek Mukherjee, Raisa Ghosh

**Affiliations:** 1 Internal Medicine, Rutgers Robert Wood Johnson Medical School/Saint Peter's University Hospital, New Brunswick, USA

**Keywords:** spontaneous, rectus sheath, abscess, drug abuse, intravenous drug user

## Abstract

Intravenous drug use has become a worldwide public health hazard and continues to affect all strata of our society. It has been associated with a multitude of severe infectious complications, such as hepatitis B, hepatitis C, human immunodeficiency virus, and endocarditis, though others such as skin and soft tissue infections are also extremely common. Rectus sheath abscess remains a rare medical condition. Here we report a 62-year-old man, who used heroin daily, presenting with an abdominal swelling with pain for two weeks. CT of the abdomen revealed a large left-sided rectus sheath abscess. Intravenous antibiotics were started and the abscess drained. The patient responded favorably to treatment. While managing complications of injection drug abuse, apart from the medical management, interventions to treat addiction assume prime importance. Rehabilitation, needle exchanges, and injection hygiene remain key to battling this malady.

## Introduction

Intravenous drug use (IVDU) is a modern public health peril and has engulfed the world [1]. It has been associated with life-threatening endocarditis and other systemic infections [2]. Often IVDU is associated with injection site infectious complications as well as other skin and soft tissue infections [3]. Rectus sheath abscess (RSA) is uncommon and is sporadically seen as a complication of rectus sheath hematoma (RSH) [4]. It has however not been reported before as a likely complication of intravenous drug abuse. Here we report the case of a 62-year-old man with a history of intravenous heroin use who presented with a painful abdominal swelling and was diagnosed with an RSA. He was treated with antibiotics and drainage of the abscess. He has recovered fully thereafter.

## Case presentation

A 62-year-old homeless man, who was a regular intravenous heroin user, presented with a gradually increasing abdominal swelling with pain for two weeks. He reported occasional chills, but no fever, chest pain, palpitations, dyspnea, or eruptions in palms or soles. He did not report any recent trauma or instrumentation in his abdomen and informed that he has only used the antecubital veins for intravenous heroin use. The patient further informed that the abdominal swelling had burst a few days ago with the discharge of pus.

**Figure 1:**
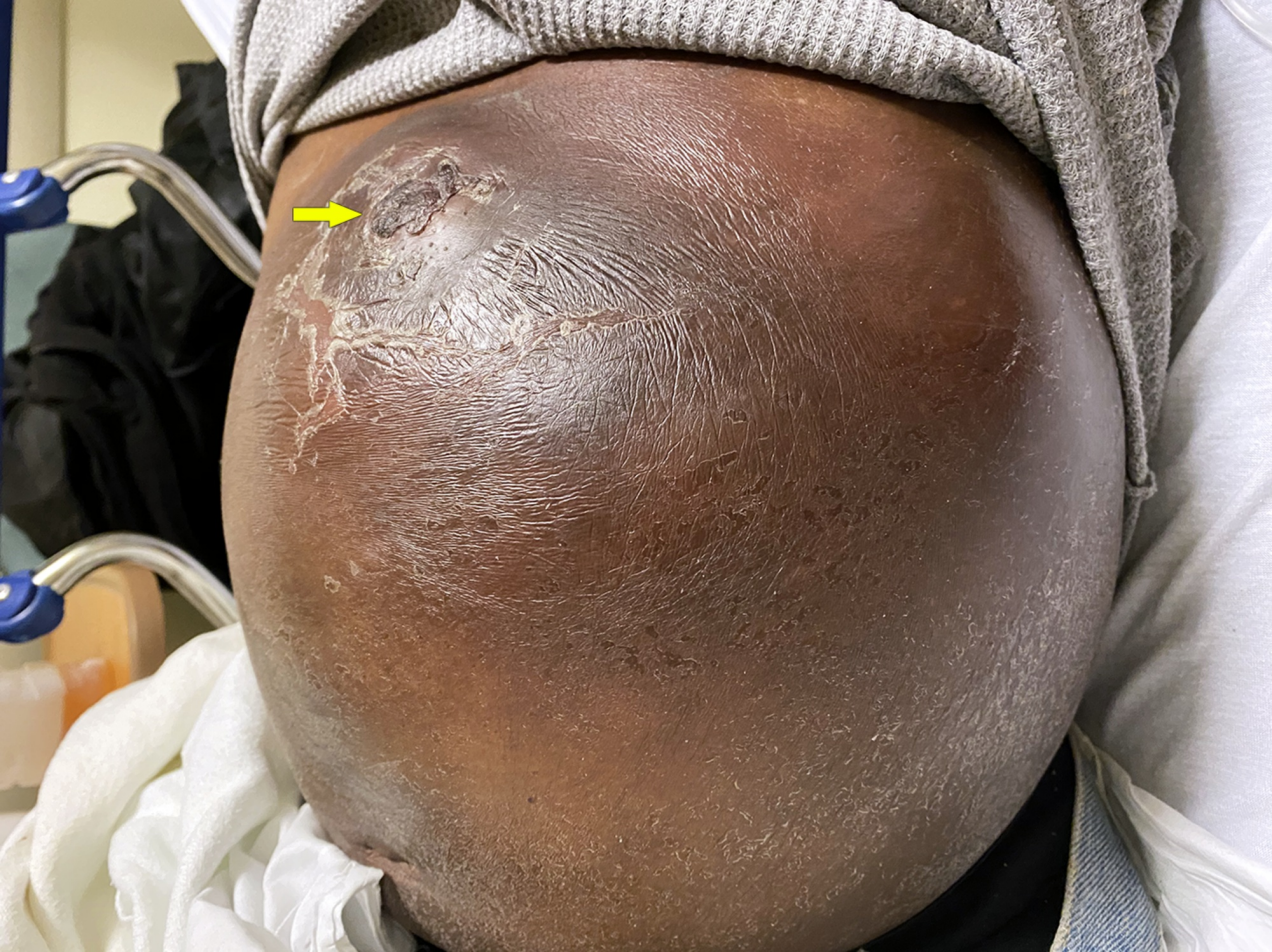
Large, left-sided abdominal wall swelling, with a recently healed wound (arrow).

At presentation, vital signs showed that he was afebrile, tachycardic at 105 beats/minute, hypotensive with blood pressure 90/50 mmHg with a normal respiratory rate (14/minute), and saturation (98%) on room air. Examination showed a distended abdomen with a tender, erythematous, and fluctuant swelling about 20 cm x 15 cm on the left anterior abdominal wall with a recently healed wound (Figure 1).

Carnett’s sign was positive, as evidenced by worsened abdominal pain with bilateral straight leg raising. Bilateral antecubital fossae showed numerous needle track marks without any abscesses. A cardiovascular examination did not reveal any murmurs.

**Figure 2:**
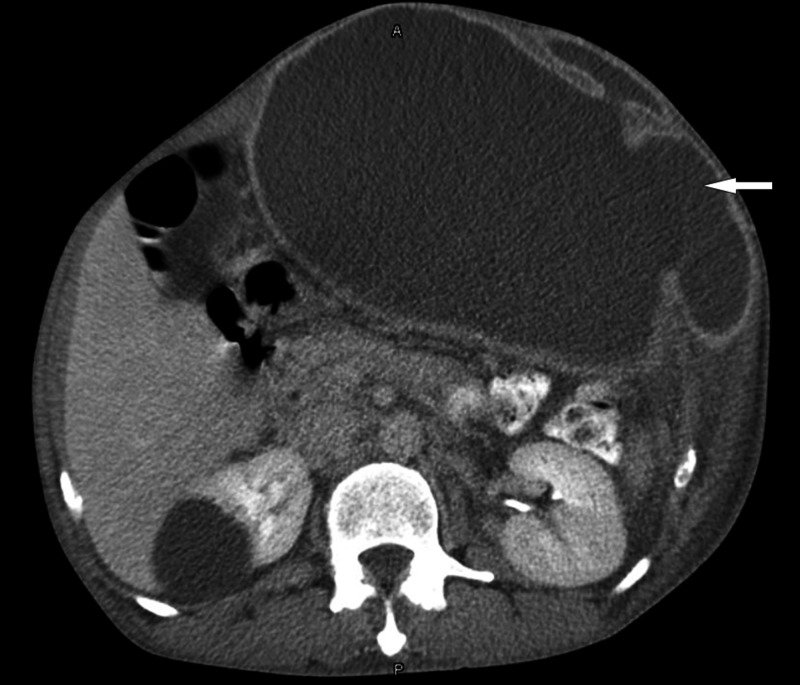
CT of the abdomen showing a left-sided rectus sheath abscess (arrow).

Investigations revealed an elevated white blood cell (WBC) count of 12 x 10^9^/L (normal: 4-11 x 10^9^/L) and C-reactive protein (CRP) at 30 mg/L (normal <5 mg/L), with normal lactic acid of 1.6 mmol/L (up to 2 mmol/L is normal). Echocardiogram revealed a normal study without any vegetations. CT of the abdomen showed a large left-sided rectus sheath collection depicting the abscess (Figure 2).

Fluid resuscitation was initiated with bolus intravenous hydration in the setting of suspected sepsis, followed by a maintenance intravenous fluid regimen. Empiric intravenous broad-spectrum antimicrobial coverage with vancomycin and piperacillin-tazobactam was initiated. After the initial stabilization of the patient, ultrasound-guided aspiration of the abscess was undertaken, which immediately drained 1,200 mL of viscous purulent fluid. An indwelling drain was left in situ to evacuate the remaining intra-abdominal fluid collection. The aspirate was sent for culture, revealing *Enterococcus faecalis* and *Enterobacter cloacae* in the growth. However, blood cultures showed no growth even after five days. Antibiotics were de-escalated to piperacillin-tazobactum based on the culture sensitivity pattern and continued for the next few days. Within the first four days, the indwelling drain further collected four liters of purulent fluid, before it was discontinued upon the resolution of the abscess. The patient recovered with the resolution of the abscess and his WBC count and CRP normalized to 7 x 10^9^/L and 4 mg/L, respectively. A week later, he was discharged on oral amoxicillin-clavulanic acid. We provided social support resources to the patient and had ongoing rehabilitation counseling before discharge. Unfortunately, the patient did not follow up with his subsequent appointments.

## Discussion

IVDU has become a worldwide public health hazard and continues to affect all strata of our society [1]. Recent data show that it affects the majority of the population in the 15-64 years age group and especially people in the lower socioeconomic strata [1]. Intravenous drug abuse is associated with a multitude of severe infectious complications, such as hepatitis B, hepatitis C, human immunodeficiency virus (HIV) and endocarditis, though other infectious complications are also very common [2,3,5].

RSA may rarely be seen as a complication of RSH [4]. It has been reported as a complication of draining a RSH [6]. RSA was also reported in a patient with normal vaginal delivery as a complication of local cellulitis [7]. RSH is an uncommon occurrence that results from an injury to the epigastric artery or its branches within the rectus abdominis muscle, leading to the formation of a hematoma within the muscle body [8-10]. The hematoma might also develop as a complication of abdominal surgeries and in chronic kidney disease [8,10]. Both RSH and RSA are associated with significant morbidity and mortality [11].

Previous studies performed in San Francisco, Baltimore, and Boston have identified a high prevalence of skin and soft tissue infections and chronic wounds in intravenous drug users [12-14]. The usage of drug paraphernalia and equipment, source of the drugs, and method of administration all have a critical role as risk factors for IVDU-related infections [2,3,5]. Adulterated drugs and speed balling, which involves mixing vasoconstricting drugs (cocaine or methamphetamine) with heroin before injection, increases the risk of infection [5,12,13]. Other injection techniques, such as skin popping (subcutaneous and intramuscular injections) and booting, also known as kicking or jacking, which involves aspiration of venous blood into the syringe followed by repeated flushing, are high-risk factors for infections [5,12-14]. Poor skin hygiene and use of dirty or shared needles are independent risk factors as well [5,12-14]. RSA in IVDU may develop via numerous mechanisms, such as bacteremia, septic embolization from underlying endocarditis, contiguous local spread, or even direct inoculation into a RSH while skin popping [5,15]. Chronic underlying conditions such as malnutrition, diabetes mellitus, HIV, and cancer can further predispose to indolent infections like RSA by impairing host defense and increasing susceptibility to infections [4,5]. The responsible organisms are often skin commensals, and the infection can occasionally be polymicrobial [5]. In our patient, we hypothesized that the RSA possibly developed from bacteremia in the absence of endocarditis and without other high-risk injection practices.

The diagnosis of RSA is generally clinical; however, radiological studies including ultrasound and CT are routinely employed [4,11,15]. Cultures are a key adjunct for judicious antimicrobial therapy [4,15]. The most crucial step for the management of an infection, especially an abscess, is rapid and adequate source control. It may be undertaken via surgical or radiologically guided abscess drainage under cover of broad-spectrum antimicrobials, which may further be adjusted according to the antimicrobial sensitivity [4,5,15]. However, in a patient with drug abuse, apart from medical management, perhaps the most critical intervention is social. De-addiction or reducing drug use, needle exchanges, and injection hygiene are key for prevention [2,5,13,15]. Addressing this social issue of drug abuse might help to heal the patient as a whole and ensure their continued well-being.

## Conclusions

RSA remains an uncommon condition. Due to its association with significant morbidity and mortality, physicians should be aware of this rare condition for its effective management. When presenting as a complication of drug abuse, apart from medical management, interventions to treat addiction assume prime importance. Rehabilitation, needle exchanges, and injection hygiene remain key to battling this malady.
